# Effect of Sugarcane Bagasse-Derived Cellulose Nanocrystals on the Thermal, Structural, Morphological and Biodegradation Properties of Poly(ε-caprolactone) and Poly(lactic Acid)

**DOI:** 10.3390/polym18091132

**Published:** 2026-05-04

**Authors:** Mbongeni Ngwenya, Thandi Patricia Gumede, Bennie Motloung

**Affiliations:** 1 Department of Life Sciences, Central University of Technology, Bloemfontein 9301, Free State, South Africa; mbongeningwenya96@gmail.com; 2 Department of Chemistry and Polymer Science, Stellenbosch University, Private Bag X1, Matieland 7602, South Africa

**Keywords:** poly(ε-caprolactone), poly(lactic acid), nanocellulose, bionanocomposites, crystallinity, morphology, sustainability

## Abstract

Biodegradable materials offer promising alternatives to petroleum-based polymers. This study investigates poly(ε-caprolactone) (PCL) and poly(lactic acid) (PLA) nanocomposites reinforced with 1, 3 and 5 wt.% cellulose nanocrystals (CNCs) extracted from sugarcane bagasse via melt blending. The thermal, structural, morphological and biodegradation properties were evaluated using differential scanning calorimetry (DSC), scanning electron microscopy (SEM), X-ray scattering (WAXS/SAXS), Fourier-transform infrared spectroscopy (FTIR), thermogravimetric analysis (TGA) and biodegradation tests. SEM results revealed uniform dispersion of CNCs at low concentrations, whereas agglomeration occurred at higher concentrations for both PCL and PLA. At 1 wt.% CNCs, there was minimal impact on the biodegradation rates of both polymers, despite achieving uniform dispersion. However, significant acceleration in biodegradation was observed at 5 wt.% CNCs, attributed to the enhanced hydrophilic nature of the nanocomposites. CNCs acted as nucleating agents in PCL crystallization, while reducing the crystallization rate of PLA. This led to a mass loss of 36.4% for PCL and 82.2% for PLA, correlating with increased and decreased crystallinities, respectively. The study concludes that the hydrophilic–hydrophobic balance has a more significant influence on biodegradation rates than crystallinity or CNC dispersion.

## 1. Introduction

The global demand for sustainable and biodegradable materials in packaging and biomedical applications has driven significant interest in biopolymer systems with tunable mechanical performance, thermal stability, interfacial compatibility and controlled biodegradation behavior, particularly those based on poly(ε-caprolactone) (PCL) and poly(lactic acid) (PLA). Despite being a synthetic polymer, PCL offers biodegradable characteristics and is a promising alternative to conventional petroleum-based plastics while PLA is derived from renewable resources such as corn starch, providing degradation under specific conditions [1]. PCL exhibits excellent flexibility and thermal stability but degrades slowly and has low mechanical strength [2]. In contrast, PLA is known for its high tensile strength and biodegradability yet it suffers from brittleness and low thermal stability [3]. A growing body of research has shown that the incorporation of nanoscale reinforcements, such as cellulose nanocrystals (CNCs), can further enhance the performance of biopolymer matrices. CNCs are rod-like crystalline domains derived from native cellulose, characterized by their large surface area, high crystallinity, mechanical strength, strong hydrogen bonding capability and biodegradability. Among the various lignocellulosic biomass sources, sugarcane bagasse (SCB), an abundant agricultural by-product, offers a cost-effective and sustainable alternative to conventional wood-based sources for the production of CNCs [4,5]. Although the properties of CNCs are strongly dependent on their source and extraction method, SCB-derived CNCs (SCB-CNCs) exhibit characteristics that can differ from those obtained from other lignocellulosic sources, including variations in particle dimensions, aspect ratio and potential surface chemistry due to the heterogeneous nature of the raw biomass and processing conditions. In particular, the presence of residual non-cellulosic components (i.e., lignin, hemicellulose, etc.) and differences in surface functional groups may influence their dispersion within polymer matrices and interfacial interactions with PLA and PCL. These factors can ultimately affect the thermal stability, crystallization behavior and biodegradation of the resulting nanocomposites. CNCs extracted from SCB not only contribute to value-added waste utilization but also serve as excellent nanofillers due to their relative compatibility with biopolymer matrices [6,7,8,9,10].

Incorporating CNCs into PCL and PLA matrices has been shown to enhance key material properties, including thermal behavior, crystallinity, barrier properties, morphology, mechanical strength, stiffness and biodegradability [11]. The degree of these effects depends on factors such as CNCs source and dispersion, interfacial interaction and nanofiller loading. For example, PCL/CNCs prepared by solution casting demonstrate promising enhancements in tensile modulus, biodegradability and moisture barrier behavior [12]. Similarly, PLA/CNCs composites exhibit improvements in mechanical, thermal and barrier properties, tailored for applications such as packaging, medical engineering and automotive industries [7,8,13]. Despite these prior studies, few investigations have systematically evaluated the effect of SCB-derived CNCs (SCB-CNCs) on both PCL and PLA simultaneously, particularly with respect to their thermal, structural, morphological and biodegradation behavior under identical processing and characterization conditions. In this context, the evaluation of thermal, structural, morphological and biodegradation properties is essential in establishing structure–property relationships in PCL/CNCs and PLA/CNCs nanocomposites. Thermal analyses provide insights into processing conditions and material stability, while crystallinity and phase behavior govern mechanical performance and degradation kinetics. Morphological analysis is critical for understanding the dispersion of CNCs within PCL and PLA and the interfacial compatibility between phases, which directly influence nanocomposite performance. Lastly, biodegradation studies are necessary to assess the environmental impact and functional lifetime of these materials. Together, the above properties provide a comprehensive understanding of the suitability of PCL/CNCs and PLA/CNCs nanocomposites for sustainable applications.

Although the impact of CNCs and their surface chemistry on the thermal, structural, morphological, mechanical and biodegradation properties of PCL and PLA has been extensively studied, to the best of our knowledge, no research has systematically investigated the relationship between the degree of crystallinity of the polyester matrices, the dispersion and concentration of SCB-CNCs, as well as the resulting biodegradation rates of PCL and PLA, particularly analyzed side by side. While previous studies have highlighted the importance of CNCs dispersion in influencing the biodegradation rates of these polyesters, our findings demonstrate that the hydrophilic–hydrophobic balance plays a more pivotal role. This is true even when CNCs serve as nucleating agents, enhancing the crystallinity of the polymer matrices, which is known to slow down the biodegradation rate.

## 2. Materials and Methods

### 2.1. Materials

Poly(ε-caprolactone)

PCL granules were purchased from 2M BIOTEC LLP, 180 Annal Gandhiadigal Salai, Ramanathapuram 623501, TN, India. It has a glass transition temperature (*T_g_*) of −60 °C, a melting temperature (*T_m_*) of 40–60 °C, and a density of 1.10 g/cm^3^.

Poly(lactic acid)

PLA granules were purchased from 2M BIOTEC LLP, 180 Annal Gandhiadigal Salai, Ramanathapuram 623501, TN, India. It has a glass transition temperature (*T_g_*) of 55–60 °C, a melting temperature (*T_m_*) of 145–155 °C, and a density of 1.23 g/cm^3^.

Cellulose nanocrystals (CNCs) were obtained from sugarcane bagasse; the employed extraction process is described in Section 2.2.

Sodium chlorite (Sigma-Aldrich, >80%), sodium hydroxide (Sigma-Aldrich, >98%), toluene (Sigma-Aldrich, >99%), ethanol (Sigma-Aldrich, >99%), glacial acetic acid (Merck, 100%) and sulfuric acid (Merck, 95–97%) were used as received.

### 2.2. Cellulose and Cellulose Nanocrystals Extraction

*Saccharum officinarum*, commonly known as sugarcane (Figure S1a), is a grass species that belongs to the family Poaceae, class Liliopsida, and order Poales. It is a globally cultivated cash crop and the primary feedstock for sugar production [13,14,15]. After sugar extraction through milling, the remaining fibrous residues are known as bagasse (Figure S1b). In this study, dry and ground bagasse was supplied by a farm in KwaZulu-Natal, South Africa. It typically comprises approximately 40–50% cellulose, 25–35% hemicellulose and 5–10% lignin [16,17]. The focus of this study was on cellulose because of its high tensile strength, biodegradability, biocompatibility and chemical modifiability characteristics in line with those of the PCL and PLA, making it a key interest in green composite development [18,19,20].

Cellulose extraction:

Figure S1 illustrates the process of extracting CNCs from SCB. Ground sugarcane bagasse (40 g dry mass) (Figure S1b) was screened to a 30-mesh particle size, and de-waxed using a 2:1 (*v*/*v*) toluene:ethanol mixture for six hours to remove surface waxes and extractives. The de-waxed bagasse (Figure S1c) was then treated with 800 mL of distilled water at 80 °C for two hours to remove soluble impurities. Delignification was achieved using a 1.3% sodium chlorite solution, maintaining pH between 3.5 and 4.0 with 6 M acetic acid, repeated until a cream white product was obtained (Figure S1d). The delignified fibers were further treated with 2% NaOH for two hours, followed by 5% NaOH at 90 °C for two hours. The residue was filtered, washed to a neutral pH, and oven-dried at 105 °C for six hours (Figure S1e) [17,21].

Cellulose nanocrystals extraction:

The dried cellulose (Figure S1e) was subjected to acid hydrolysis using 50% H_2_SO_4_ at a cellulose-to-acid ratio of 1:10 g/mL at 45 °C for one hour, with constant stirring (5000 rpm) using a homogenizer (T25 Digital Ultra-Turrax, IKA, Staufen im Breisgau, Germany). To terminate the reaction, 10-fold ice-cold distilled water was added, followed by centrifugation at 10,000 rpm for 15 min (three cycles, at 10 °C). The collected sediment was dialyzed against distilled water for ~48 h until a neutral pH was achieved. The resulting suspension (5 mg/mL) was sonicated for 10 min in an ice bath to prevent overheating. The resulting CNCs (Figure S1f) were obtained by freeze-drying the suspension (0.2 wt.% in distilled water) at reduced pressure and low temperature, producing a light brownish fine powder while preserving particle integrity and porosity [22,23,24]. Table S1 presents the percentage yield of cellulose and CNCs. From 40 g of sugarcane bagasse, 10.5 g of cellulose was obtained (26.3% yield relative to SCB), which was further converted into 7.7 g of CNCs (19.25% yield relative to SCB). These values indicate a decent extraction output, consistent with what is reported in the literature [22,25]. The yield provided sufficient CNCs for subsequent incorporation into PCL and PLA. Table 1 summarizes selected plant-based sources of CNCs, together with their yields and resultant properties. The yields ranged from 7 to 22% relative to starting fiber material, with the exception of jute and teff straw, which recorded generally higher values, albeit relative to the extracted cellulose. The high yield observed for bleached cotton is due to the inherently high cellulose content of the plant. The CNCs’ diameter ranged from 6 to 55 nm, which translates to high aspect ratio, while the crystallinity was mostly above 70%.

### 2.3. Composite Production

Table S2 summarizes the drying conditions used to remove residual moisture from PCL, PLA and CNCs before melt blending. PCL was dried at 40 °C while PLA and CNCs were dried at 50 °C, all under vacuum overnight. Proper drying at these controlled temperatures is important for preventing hydrolytic degradation during processing, as both PCL and PLA are moisture sensitive at elevated temperatures, while CNCs readily retain bound water due to their hydroxyl-rich surfaces. Ensuring consistent drying therefore improved process reproducibility and minimized variations in the resulting thermal and morphological properties.

Table 2 shows the compositions of PCL, PLA and CNCs in the nanocomposites prepared in this study. CNCs were incorporated into each polymer at 1, 3, and 5 wt.% to investigate the effects of filler concentration, with 1 wt.% representing a low content, 3 wt.% an intermediate level and 5 wt.% a high loading where agglomeration might occur. Per composition, a total of 40 g of the combined materials (i.e., PCL, PLA and CNCs) were weighed and processed to produce a continuous film of ca. 2 mm thickness. Melt blending was carried out using a Brabender Plastograph (Brabender GmbH & Co, Kulturstraße, DU, Germany) at 180 °C and 50 rpm for 12 min. The blended samples were allowed to cool at room temperature overnight for recrystallization, and the solidified materials were then compression-molded using a hydraulic press at 180 °C for five minutes under 50 kPa pressure. This systematic variation in CNCs concentration allowed for the correlation of filler content with the thermal, structural and biodegradation performance of the resulting nanocomposites.

### 2.4. Characterization of Composites

Differential scanning calorimetry

Differential scanning calorimetry (DSC) was performed using PerkinElmer DSC 6000 (Waltham, MA, USA). The samples (~6.0 mg) were heated from 0 to 180 °C (first heating scan), held at this temperature for three minutes to erase the thermal history, then cooled from 180 to 0 °C (cooling scan), held for one minute at 0 °C for conditioning, and finally heated from 0 to 180 °C (second heating scan). The scanning rate in all steps was 10 °C per minute, and the experiments were conducted using a 20 mL per minute nitrogen flow. Cooling and second heating scans were used to determine thermal transitions. The focus was on parameters such as crystallization temperature (*T_c_*), glass transition temperature (*T_g_*), cold crystallization temperature (*T_cc_*), melting temperature (*T_m_*), enthalpies (*∆H*) and degree of crystallinity (*X_c_*), which was calculated using Equation (1). These parameters provided insights into the interactions between PCL and CNCs, as well as PLA and CNCs.

(1) $$X_{c}=\left(\frac{{\Delta H}_{m}-{\Delta H}_{cc}}{w{∆H}_{m^{o}}}\right)\times 100\%$$

where *X_c_* is the degree of crystallinity, *∆H_m_* is the melting enthalpy of the measured sample, *∆H_cc_* is the cold crystallization enthalpy of the measured sample, *∆H_m_^o^* is the melting enthalpy of 100% crystalline polymer, and *w* is the weight fraction of a polymer in the blend or composite. The values of *∆H_m_^o^* used for PCL and PLA are 139 J/g and 93.7 J/g, respectively [1,35].

Scanning electron microscopy

Samples were cryo-fractured, mounted on aluminum pin stubs using epoxy glue, and sputter-coated with Iridium using a Leica EM ACE600 (Leica Microsystems GmbH, Wetzlar, Germany). Imaging was performed on a JEOL JSM-7800F extreme-resolution analytical field emission SEM (Tokyo, Japan), at 5 kV, 10 mm working distance.

X-ray scattering analysis

Simultaneous small-angle X-ray scattering/wide-angle X-ray scattering (SAXS/WAXS) was performed at beamline BL11-NCD, ALBA Synchrotron, Barcelona, Spain. Samples were placed in DSC pans on a Linkam THMS600 hot stage (Tadworth, UK), cooled/heated via a liquid nitrogen system. The thermal protocol involved: heat 20 °C per minute from 25 to 180 °C, hold for three minutes at 180 °C, cool 20 °C per minute from 180 °C to 0 °C, hold for one minute min at 0 °C, heat 20 °C per minute from 0 to 180 °C, thus reproducing the crystallization conditions employed in the DSC experiments. The X-ray energy was 12.4 keV (ʎ = 1.0 Å). The reported data was taken from cooling and heating ramps at selected temperatures. The WAXS/SAXS data were taken simultaneously, using the ADSC Q315r detector (Malvern Panalytical, Malvern, Worcestershire, UK), 3070 × 3070 pixels, pixel size 102 μm^2^, distance 6495 mm, tilt angle 0° for SAXS, and the Rayonix LX255-HS detector (Rayonix LLC, Evanston, IL, USA), 1920 × 5760 pixels, pixel size 44 μm^2^, distance 132.6 mm, tilt angle 21.2° for WAXS. These detectors were calibrated using the following standards: silver behenate (SAXS) and Cr_2_O_3_ (WAXS). Scattering intensity versus scattering vector (*q* = 4*πsinθ*/*λ*) was analyzed. The WAXS patterns (i.e., intensity (*I*) vs. scattering vector (*q*)) provide insight into the crystalline structures of neat polymers (i.e., PCL and PLA), neat CNCs, and their nanocomposites. The crystallinity ($X_{cx}$) was calculated using Equation (2):

(2) $$X_{cx}=\frac{I_{c}}{I_{c}+\;I_{a}}$$

where $I_{c}$ and $I_{a}$ are respectively the integrated intensities of the crystalline and amorphous phases. For SAXS, the long period (*L*), representing the average spacing of lamellar structures, was calculated using Equation (3), and lamellar thickness ($l_{c}$) was calculated using Equation (4):

(3) $$L=\frac{2\pi }{q_{max}}$$

where $q_{max}$ is the position of the peak maximum in nm^−1^.

(4) $$l_{c}=L.X_{cx}$$

where L is the long period in nm and $X_{cx}$ is the crystallinity.

Fourier transform infrared spectroscopy.

Fourier transform infrared spectroscopy (FTIR) spectra were obtained using a PerkinElmer Spectrum 100 FTIR spectrometer (Waltham, MA, USA) equipped with a PIKE MiracleTM (PIKE Technologies Inc, Madison, Wisconsin, USA) attenuated total reflectance, with a diamond crystal. Background scans were collected before each measurement. Scans were recorded over a range of 4000 and 650 cm^−1^, at 8 cm^−1^ resolution, with eight scans per sample.

Thermogravimetric analysis

Thermogravimetric analysis (TGA) was carried out using PerkinElmer STA 6000 (Waltham, MA, USA) under 20 mL per minute nitrogen. Samples (~23 mg) were heated from 30 to 600 °C at a rate of 10 °C per minute. The collected data was analyzed using Pyris software (version 11).

Biodegradation

Aerobic biodegradability was assessed by cutting small specimens (ca. 2 cm × 2 cm) of each sample and burying them in soil (4 cm deep, 39% moisture) at room temperature for 112 days following ASTM D5988 [36]. However, variations in shape occurred due to the brittle nature of some samples. At 14-day intervals, samples were retrieved, washed with distilled water, dried in an oven at 30 °C for one hour, and weighed. Photographs were taken at each interval to visually monitor the progress of biodegradation.

## 3. Results and Discussion

### 3.1. Thermal Properties

**Figure 1:**
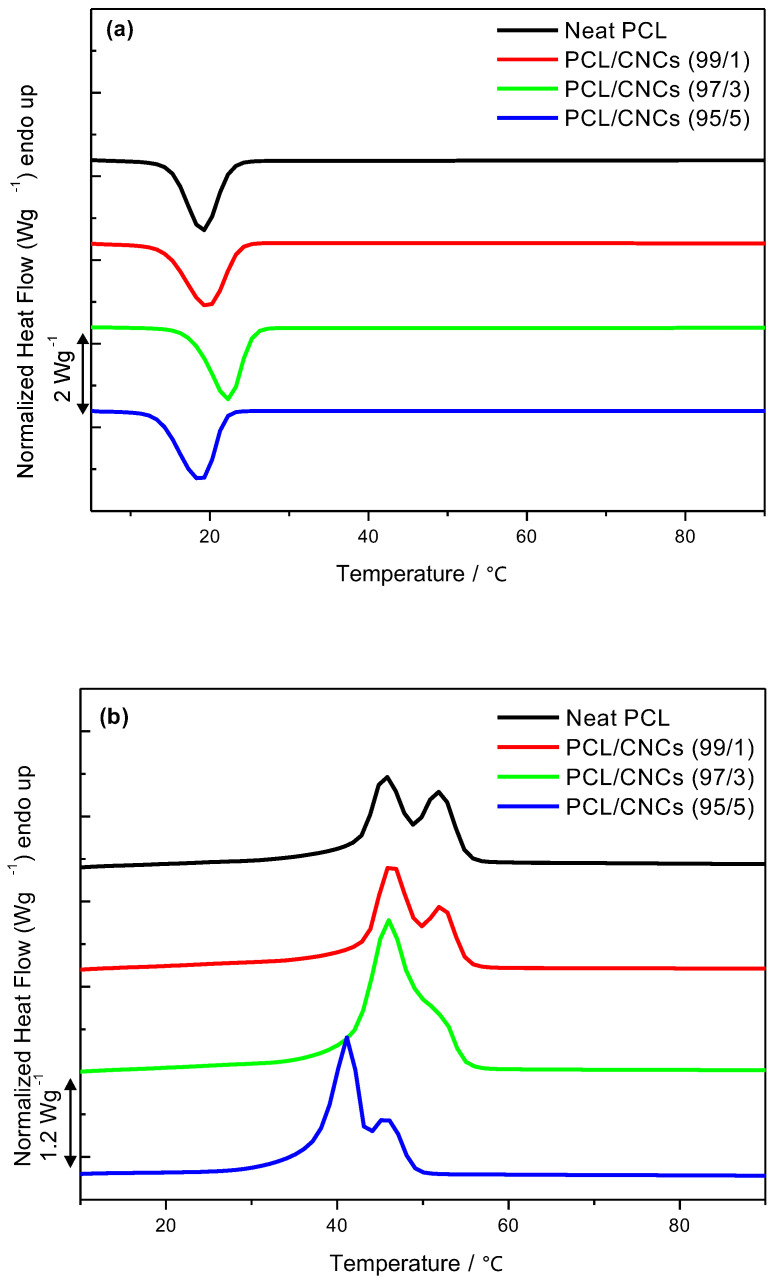
Differential scanning calorimetry curves: (**a**) cooling curves for neat PCL and PCL/CNCs nanocomposites, and (**b**) second heating curves for neat PCL and PCL/CNCs nanocomposites containing 1, 3 and 5 wt.% CNCs.

**Figure 2:**
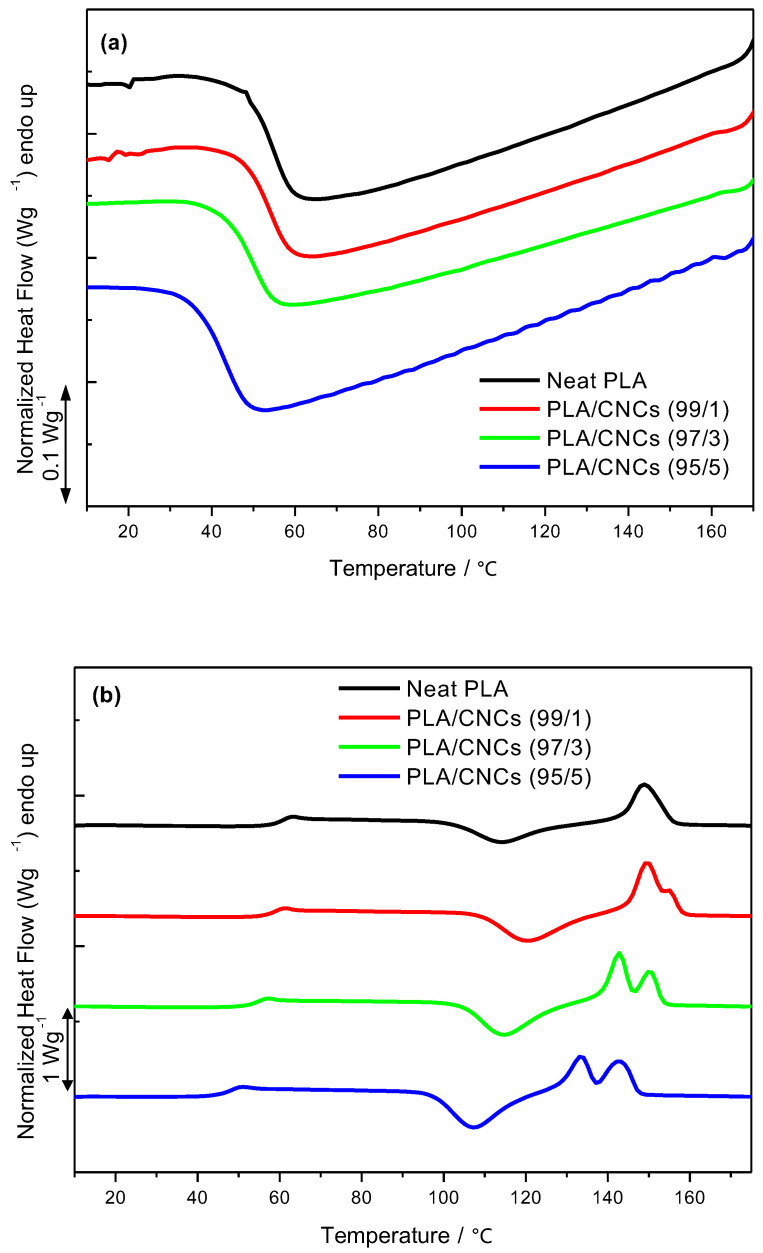
Differential scanning calorimetry curves: (**a**) cooling curves for neat PLA and PLA/CNCs nanocomposites, and (**b**) second heating curves for neat PLA and PLA/CNCs nanocomposites containing 1, 3, and 5 wt.% CNCs.

DSC results for neat PCL, PLA and their nanocomposites with 1, 3, and 5 wt.% CNCs are presented in Figure 1 and Figure 2 and Table 3 and Table 4.

Figure 1a,b present the cooling and second heating scans for neat PCL and PCL/CNC nanocomposites with 1, 3 and 5 wt.% CNCs loading. Neat PCL shows two distinct melting peaks at 45.6 °C and 52.2 °C, suggesting the coexistence of crystals with different levels of perfection, which might be due to the crystallization process [37,38]. The measured *ΔH_m_* of 56 J/g closely matches the *ΔH_c_* of 54 J/g, with a *T_c_* of 18.4 °C and the degree of crystallinity of 40.3%. For PCL/CNCs, at 1 wt.% CNCs, there is minimal impact on the thermal transitions; however, at 3 wt.%, CNCs act as effective nucleating agents, increasing *T_c_* and ordering PCL crystals into a single melting peak [6,39]. The observed nucleating efficiency can be attributed to the uniform dispersion of the nanocrystals within the PCL matrix, as well as to the strong ability of CNCs to induce heterogeneous nucleation. This behavior has been previously demonstrated by Malmström and co-workers [40], who reported a significant increase (i.e., 9.1%) in the crystallinity of PCL relative to neat PCL at a CNCs loading of 3 wt%. At higher CNCs loadings (i.e., 5 wt.%), the crystallinity of PCL remained higher than that of the neat polymer due to the continued nucleating effect of CNCs; however, the extent of this enhancement was limited. This reduction is likely due to nanocrystal agglomeration [41], which restricts polymer chain mobility and consequently attenuates the crystallization rate. Consequently, the crystallinity values at 5 wt% CNCs, while still elevated compared to neat PCL, were lower than those observed for the 3 wt% PCL/CNCs nanocomposites. This is consistent with the work done by Mi et al. [42], where only a 2% increase in the crystallinity of PCL was reported at 5 wt.% CNCs loading. The melting temperatures as a function of CNCs content are presented in the supporting information (SI) as Figure S2a.

Figure 2a,b present the cooling and the second heating scans of neat PLA and PLA/CNCs nanocomposites with 1, 3 and 5 wt.% CNCs loading. Neat PLA exhibits a *T_g_* at 59.6 °C, a *T_cc_* at 113.3 °C, and a *T_m_* at 149.1 °C, with a degree of crystallinity of 7.5%. The presence of a cold crystallization peak indicates that PLA cannot crystallize completely during cooling. PLA/CNCs (Figure 2b) show reduced *T_g_*, indicating certain flexibility of PLA chains in the presence of CNCs, due to poor interaction between hydrophilic CNCs and hydrophobic PLA. However, only 5 wt.% of CNCs can shift *T_cc_* to lower values as a nucleating agent. At all filler loadings, a *T_cc_* is detected for PLA, indicating that even in the presence of CNCs, it cannot crystallize during cooling. The presence of CNCs provokes a decrease in the *T_m_* values with increasing CNCs content (Figure S2b), which can be attributed to agglomeration problems or interactions that affect the mobility of the chains. Double melting peaks are observed at 3 and 5 wt.% CNCs loading, indicating the formation of α’- and α-form crystals due to disrupted chain alignment during blending [43,44]. The reduced crystallinity of PLA at higher CNCs loadings may be attributed to CNCs agglomeration, which can disrupt polymer chain ordering. This phenomenon also helps explain the shift in melting behavior observed in the DSC curves (Figure 2b). The presence and extent of CNCs agglomeration will be further examined in the SEM analysis presented in the following section.

**Figure 3:**
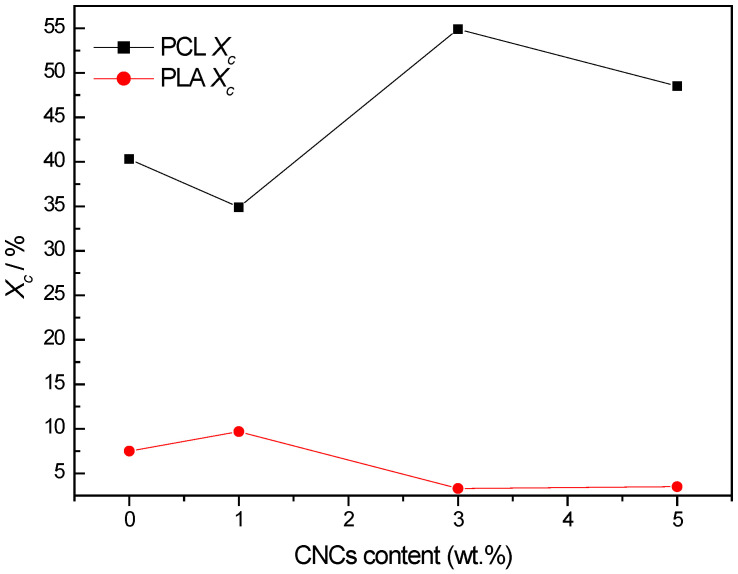
Degree of crystallinity of PCL and PLA in nanocomposites containing 1, 3 and 5 wt.% CNCs.

Figure 3 and Table 3 and Table 4 present the effect of incorporating 1, 3 and 5 wt.% CNCs on the degree of crystallinity of PCL and PLA. At low CNCs loading (i.e., 1 wt.%), the observed PCL crystallinity is lower than that of neat PCL (Table 3). This behavior may be attributed to a reduction in the crystallization rate induced by the presence of CNCs. It has been reported that the crystallization activation energy increases significantly at this CNCs loading, resulting in slower crystallization kinetics, a reduced PCL crystalline phase, and thus lower overall crystallinity compared to neat PCL [45,46]. Such an anti-nucleating effect has also been reported by Dufresne and co-workers, where CNCs were incorporated into poly(ethylene oxide) [47]. However, at higher CNCs loadings (i.e., 3 and 5 wt.%), CNCs act as effective nucleating agents, leading to an increase in the crystallinity of PCL (i.e., by 14.6 and 8.2%, respectively). However, this effect is less pronounced at 5 wt.%, likely due to nanocrystal agglomeration, *vide supra*, which restricts polymer chain mobility and partially offsets the nucleation efficiency.

In contrast, the nucleating effect of CNCs was evident at low loadings (i.e., 1 wt.%), resulting in an increase in the crystallinity of PLA (Table 4). This observation is consistent with the work of Sullivan et al. [48], who reported that CNCs act as effective nucleating agents at similar loadings. Interestingly, Ahmad et al. [49] observed a reduction in PLA crystallinity at CNC loadings of 0.75 and 1 wt.%. These contrasting findings suggest that the nucleating behavior of CNCs during PLA crystallization is not universal, but rather depends on factors such as CNC morphology, dispersion quality, and the fabrication method of the PLA/CNCs nanocomposites. At higher CNC loadings (i.e., 3 and 5 wt.%), the crystallinity of PLA in the nanocomposites decreased relative to that of neat PLA. This behavior can be attributed to the aggregation of CNCs at elevated loadings, which adversely affects their nucleation efficiency and leads to slower crystallization kinetics. Indeed, Gond et al. [50] reported uniform CNCs dispersion at 1 and 2 wt.%, whereas agglomeration was observed at loadings above 2 wt.%. Furthermore, Clarkson et al. [51] reported an increase in PLA crystallinity at 1 wt.% CNCs, while the crystallinity at 3 and 5 wt.% was lower than that of neat PLA, in agreement with the present findings.

### 3.2. Morphological Analysis

**Figure 4:**
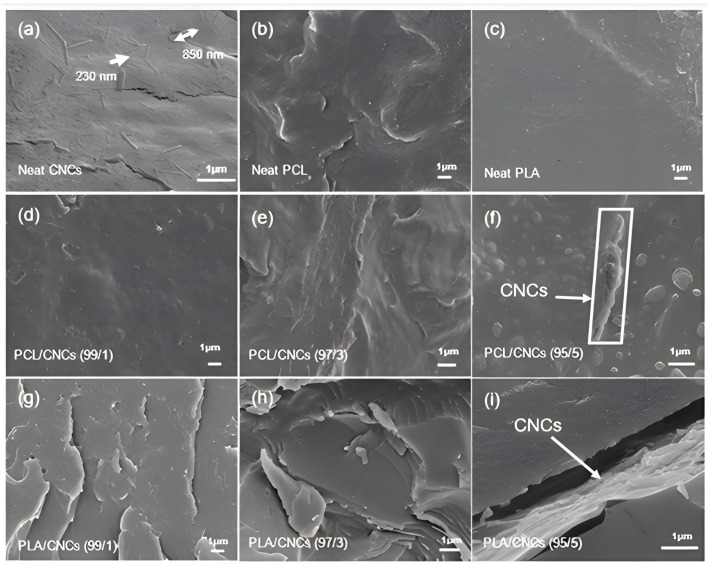
Morphological features of neat CNCs, neat PCL and PCL/CNCs nanocomposites, and neat PLA and PLA/CNCs nanocomposites with CNCs loadings of 1, 3, and 5 wt.%.

Figure 4 presents SEM micrographs illustrating the morphological characteristics of neat CNCs, neat PCL and PLA, and their nanocomposites with 1, 3 and 5 wt.% CNCs. SEM analysis provides insights into phase compatibility, dispersion and interfacial interactions of the components. Neat CNCs (Figure 4a) appear as rod-like structures with a length range from 230 nm to 850 nm, consistent with their nanoscale dimensions [25]. Neat PCL (Figure 4b), in contrast, exhibits a smooth and homogeneous surface, characteristic of its ductile and semicrystalline nature. Neat PLA (Figure 4c) displays a rough fractured surface, indicative of its brittle nature and amorphous morphology. These morphological features align with WAXS and SAXS results, where PLA showed minimal crystalline order, while PCL exhibited broader but distinct crystalline reflections.

For PCL/CNCs nanocomposites (Figure 4d–f), the fractured surface remains smooth at low CNCs content, for example, PCL/CNCs (99/1) (Figure 4d), with a slight increase in roughness due to CNCs inclusion. For PCL/CNCs (97/3), the nanocomposite morphology (Figure 4e) suggests an interaction between PCL and CNCs, as CNCs are embedded in the PCL matrix. DSC results also suggest that 3 wt.% CNCs ordered the crystals of PCL to be of the same size and increased the crystallinity of the nanocomposite. At higher CNCs loadings, for example, PCL/CNCs (95/5) (Figure 4f), surface roughness increases significantly, and CNCs aggregates are visible, reflecting reduced dispersion, which might be due to saturation.

In PLA/CNCs nanocomposites (Figure 4g–i), poor interfacial adhesion is evident, with edge formation and cracking observed on the fractured surfaces. These defects increase with CNCs content, highlighting compatibility issues between the hydrophilic CNCs and the hydrophobic PLA matrix. At 5 wt.% CNCs (Figure 4i), large CNCs agglomerates form (see the arrow). The observed CNCs agglomeration at higher loadings changes surface roughness as well as reduces dispersion uniformity. Gond et al. also reported similar observations [8,50]. These authors evaluated the properties of PLA/CNCs nanocomposites (with CNCs loadings of 1, 2, 3, 4 and 5 wt.%). They discovered that higher CNCs loadings (>2 wt.%) in the PLA matrix result in agglomeration. SEM analysis revealed that at higher CNCs loadings (5 wt.%), nanoparticles tended to agglomerate, disrupting the uniformity of the polymer matrix. The agglomeration of CNCs in PLA typically reduces the reinforcing efficiency of the nanocrystals, leading to deterioration in physical, mechanical, thermal and barrier properties of the composites due to stress concentration and poor interfacial interaction between the nanofiller and PLA matrix [8,50]. This correlates with the DSC results, where a decrease in crystallinity and a shift in the melting temperature were observed. The reduced crystallinity can thus be attributed to the presence of CNCs clusters, which hinder polymer chain ordering and act as less effective nucleation sites compared to well-dispersed nanoparticles.

### 3.3. Structural Analysis

**Figure 5:**
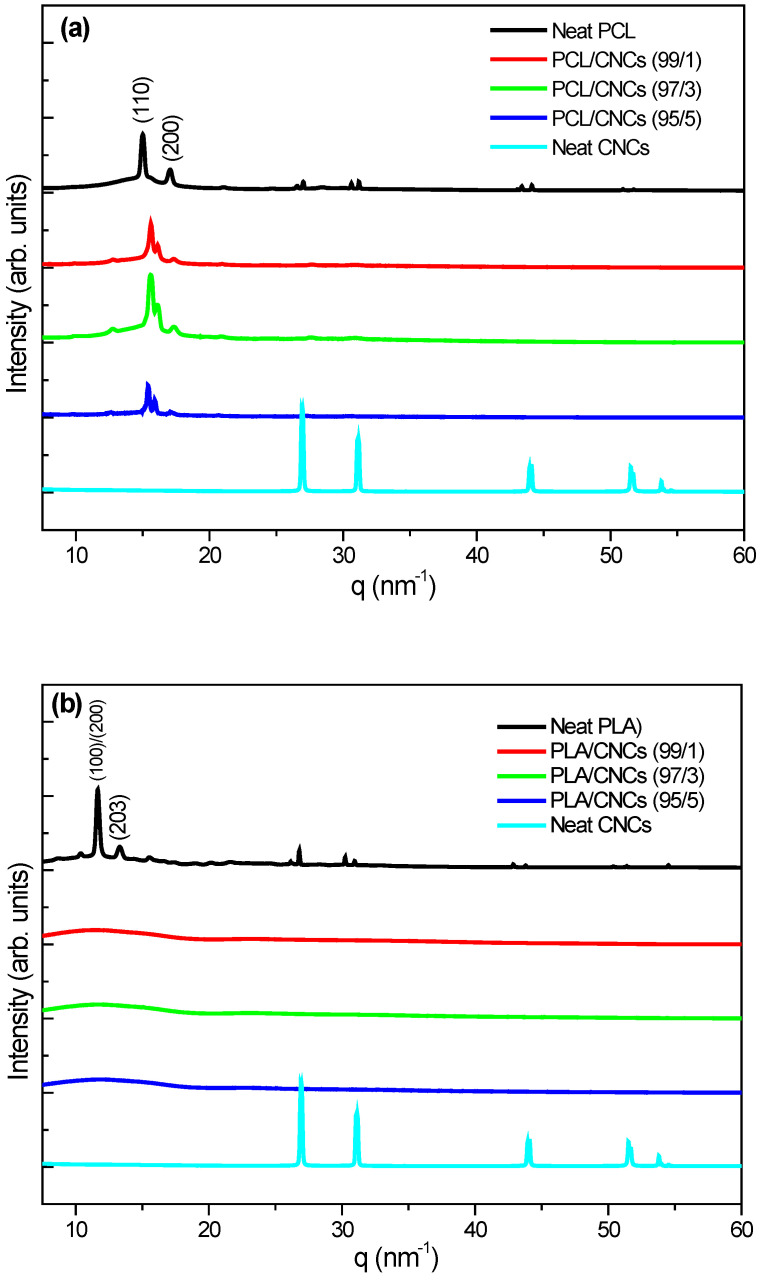
WAXS patterns of (**a**) neat PCL, neat CNCs and PCL/CNCs nanocomposites with 1, 3 and 5 wt.% CNCs, and (**b**) neat PLA, neat CNCs and PLA/CNCs nanocomposites with 1, 3 and 5 wt.% CNCs (data obtained from the second heating at 25 °C).

Figure 5a presents the WAXS spectra of neat PCL and its nanocomposites with 1, 3 and 5 wt.% CNCs. Neat PCL presents broader peaks at *q* = 15.0 and 17.0 nm^−1^, with crystallinity of 17.9% assigned to its 110 and 200 planes [52,53]. Minor peaks around 27.0 and 31.0 nm^−1^ indicate a long-range crystalline order [54]. Neat CNCs reveal sharp, intense peaks at *q* = 26.8, 31.0, 44.1, 51.2 and 53.8 nm^−1^, indicating high crystallinity (75.9%). The CNCs peaks are deviated to higher *q* values compared to the *q* values reported in literature which might be due to the source and processing of CNCs [54,55,56]. For example, Motloung et al. presented WAXS results for spray-dried CNCs with *q* = 10.6, 14.6, 15.8 and 24.1 nm^−1^ [54]. The shift is due to the decreased inter-planar spacing (*d*) in the CNCs. The decrease in *d*-spacing may be caused by thicker packing of crystals or a modification process such as drying methods [57]. PCL/CNCs nanocomposite scans show characteristics of PCL peaks with a slight shift to higher *q* values, which confirms the interaction of PCL and CNCs. At 1 wt.% CNCs loading, PCL crystallinity decreased, while it increased at 3 wt.% CNCs, which proved to be the optimum loading for improved crystallinity (Table 5). These results are in agreement with DSC results, where 3 wt.% CNCs acted as a nucleating agent and also arranged PCL crystals of the same order. However, it should be noted that crystallinity values obtained from DSC and WAXS can differ, as these techniques probe distinct aspects of the crystalline structure, with DSC often yielding higher apparent crystallinity due to thermal contributions.

Figure 5b presents WAXS patterns for neat PLA, neat CNCs, and their nanocomposites with 1, 3 and 5 wt.% CNCs. Neat PLA exhibits two sharp crystalline peaks at *q* values of 11.6 and 13.3 nm^−1^, with crystallinity of 8.9%, corresponding to the 110/200 and 203 planes of the α-form crystal structure [53,58]. The PLA/CNCs nanocomposites spectra do not show any crystal peaks either for PLA or CNCs, only a halo band from 5 to 17 nm^−1,^ which corresponds to the amorphous phase of PLA. This suggests that CNCs were unable to effectively induce crystallization during the cooling of PLA, probably due to agglomeration observed in SEM, which indicated that PLA and CNCs were not homogeneously dispersed (Figure 4i).

**Figure 6:**
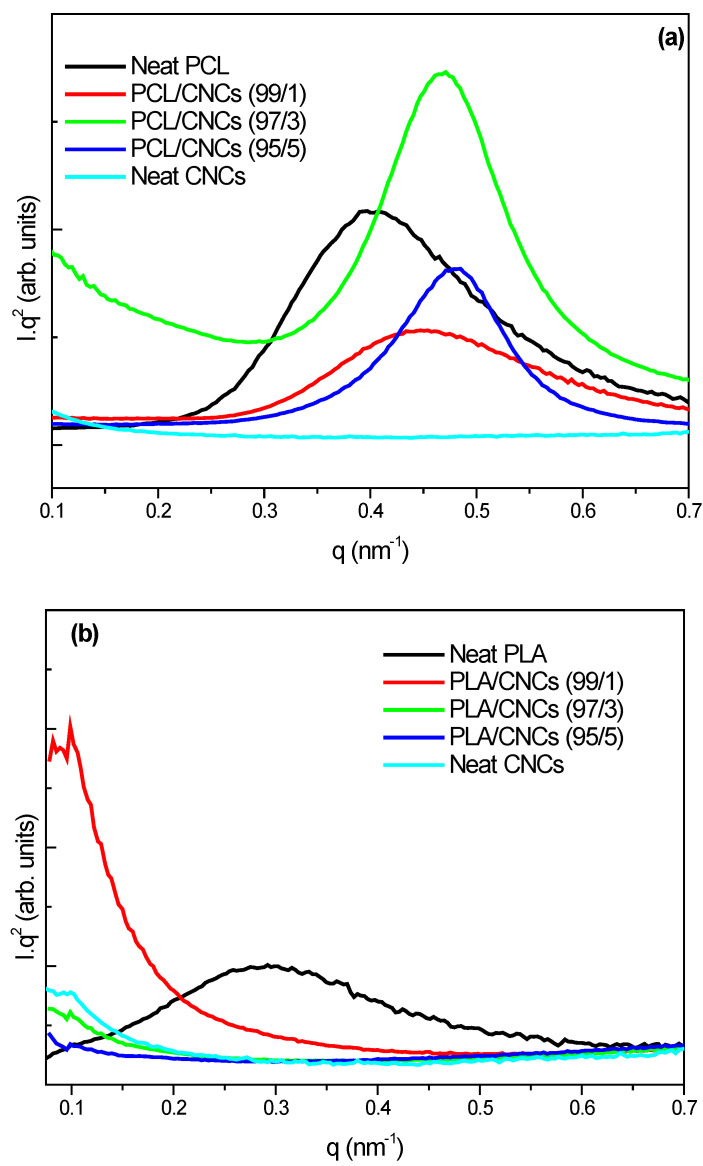
SAXS patterns of (**a**) neat PCL, neat CNCs and PCL/CNCs nanocomposites with 1, 3 and 5 wt.% CNCs, and (**b**) neat PLA, neat CNCs and PLA/CNCs nanocomposites with 1, 3, and 5 wt.% CNCs (data obtained from the second heating at 25 °C).

Figure 6 presents SAXS profiles for neat PCL, neat PLA, neat CNCs and their nanocomposites with 1, 3 and 5 wt.% CNCs loadings. SAXS provides insights into nanoscale morphology by analyzing periodicities of crystalline and amorphous layers.

Figure 6a presents SAXS patterns for neat PCL, neat CNCs and their nanocomposites with 1, 3 and 5 wt.% CNCs. Neat PCL exhibits a broader and more intense SAXS peak, with an *L* value of approximately 16.5 nm, suggesting the presence of semicrystalline lamellae with limited order [52]. Neat CNCs do not show any peak in the SAXS range due to the lack of lamellar or long-range periodicity; CNCs are single crystallites, not lamellar systems. The addition of 1 and 5 wt.% CNCs to PCL resulted in very weak SAXS peaks, while the incorporation of 3 wt.% CNCs resulted in a broad, intense SAXS peak, with an *L* value of approximately 13.7 nm, which is below that of neat PCL. This means that the spacing between crystalline lamellae (crystal + amorphous thickness) is smaller, suggesting that at 3 wt.% CNCs facilitate lamellar organization. These results are in agreement with WAXS and DSC results where it is observed that 3 wt.% CNCs improved the crystallinity of PCL. Figure 6b presents the scans of neat PLA, neat CNCs and their nanocomposites. Neat PLA and its nanocomposites show very weak or absent SAXS peaks, indicating a mostly amorphous structure, supported by the broad, less intense peaks in WAXS. SAXS profiles of intensity (*I*) as a function of scattering vector (*q*) are presented in the SI (Figure S3), and they provide similar information to that presented in Figure 6 and also summarized in Table 6.

**Figure 7:**
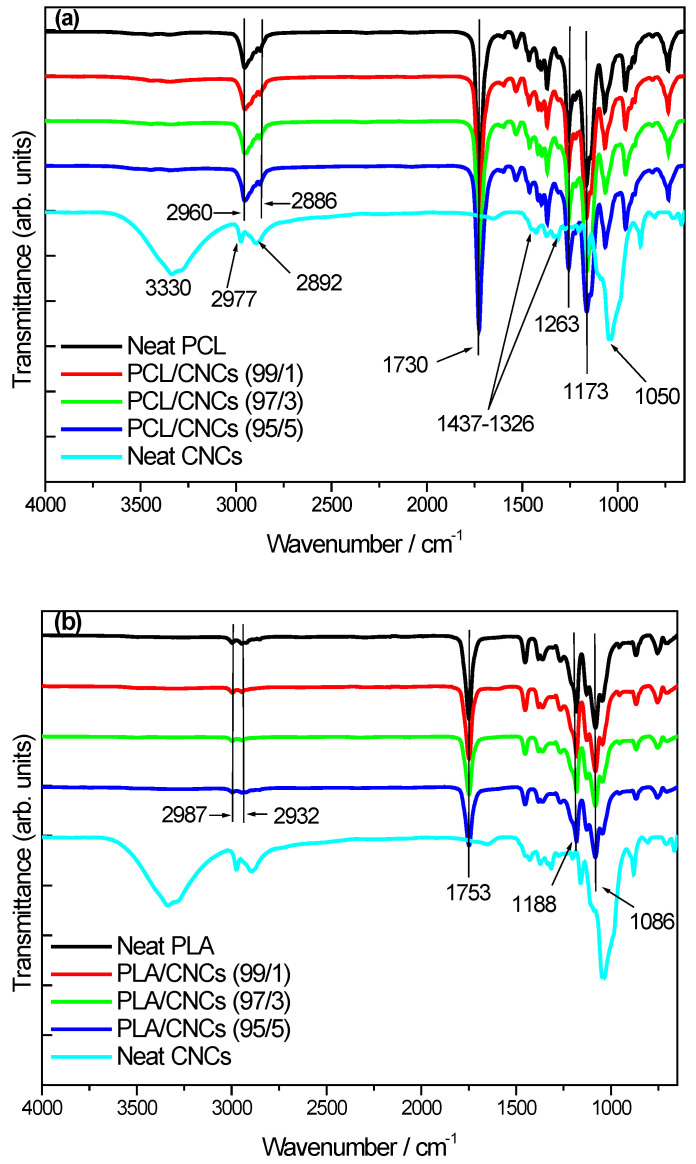
FTIR spectra for (**a**) neat PCL, neat CNCs and PCL/CNCs nanocomposites with 1, 3, and 5 wt.% CNCs; (**b**) neat PLA, neat CNCs and PLA/CNCs nanocomposites with 1, 3 and 5 wt.% CNCs.

The FTIR scans shown in Figure 7 present the characteristic functional groups of neat PCL, neat PLA, neat CNCs and nanocomposites with varying CNCs compositions. According to Figure 7a, neat PCL exhibits characteristic features, including a strong C=O stretching band at 1730 cm^−1^, and prominent C−O−C peaks at 1263 cm^−1^ (asymmetric) and 1173 cm^−1^ (symmetric). Additionally, C−H stretching vibrations are observed at 2960 and 2886 cm^−1^. The FTIR spectrum of neat CNCs (Figure 7a,b) is characterized by a broad O−H stretching band at 3330 cm^−1^, reflecting intra and intermolecular hydrogen bonding between hydroxyl groups. C−H stretching occurs at 2977–2892 cm^−1^, while the bands observed at 1437 cm^−1^ and 1326 cm^−1^ are associated with the C−H deformation and CH_2_ bending in native cellulose. The peak at 1050 cm^−1^ is attributed to C−O−C stretching and C−H rocking vibrations of the pyranose ring [19,59,60,61,62].

The FTIR spectra of the PCL/CNCs nanocomposites are presented in Figure 7a. All spectra are dominated by the characteristic absorption bands of PCL, including the ester carbonyl (C=O) stretching at ~1730 cm^−1^ and the −CH_2_ stretching vibrations in the 2800–2900 cm^−1^ region. This is expected given the relatively low CNCs content (≤5 wt%), for which the infrared response is largely governed by the PCL matrix. No new absorption bands or significant peak shifts are observed upon CNCs addition, suggesting that no new covalent bonds are formed between PCL and CNCs, consistent with physically blended systems. The characteristic O–H bands of CNCs are not clearly distinguishable, likely due to their low concentration and overlap with existing PCL absorption bands. Overall, the FTIR results suggest that the incorporation of CNCs does not significantly alter the chemical structure of PCL, and that any PCL–CNCs interactions are primarily physical in nature, as commonly reported for polymer nanocomposites at low nanofiller loadings [63,64,65].

Figure 7b presents the FTIR spectra of neat PLA, neat CNCs and PLA/CNCs nanocomposites with 1, 3 and 5 wt.% CNCs loadings. Neat PLA exhibits a medium-intensity C=O stretching peak at 1753 cm^−1^, corresponding to ester carbonyl groups. C−H stretching vibrations are observed at 2987 and 2932 cm^−1^, and C–O–C asymmetric and symmetric stretching peaks appear at approximately 1188 cm^−1^ and 1086 cm^−1^, respectively.

Similarly, in the PLA/CNCs nanocomposites, the FTIR spectra are dominated by the characteristic absorption bands of PLA, with only minor variations in intensity of the C=O stretching peak observed as the CNCs content increases. The slight reduction in band intensity observed at higher CNCs loadings (i.e., 5 wt.%) should be interpreted with caution, as the spectra were not normalized. Variations in sample thickness, baseline correction and CNCs concentration can all influence the apparent band intensities, making quantitative comparisons unreliable. No new absorption bands or significant peak shifts are detected upon CNCs incorporation, indicating that no new covalent bonds are formed between PLA and CNCs. While weak intermolecular interactions, such as hydrogen bonding between CNCs hydroxyl groups and PLA ester carbonyl groups, may be present, these effects are expected to be subtle and are not definitively resolved by FTIR at the investigated CNCs contents [8,19,50,66,67].

### 3.4. Thermal Stability

**Figure 8:**
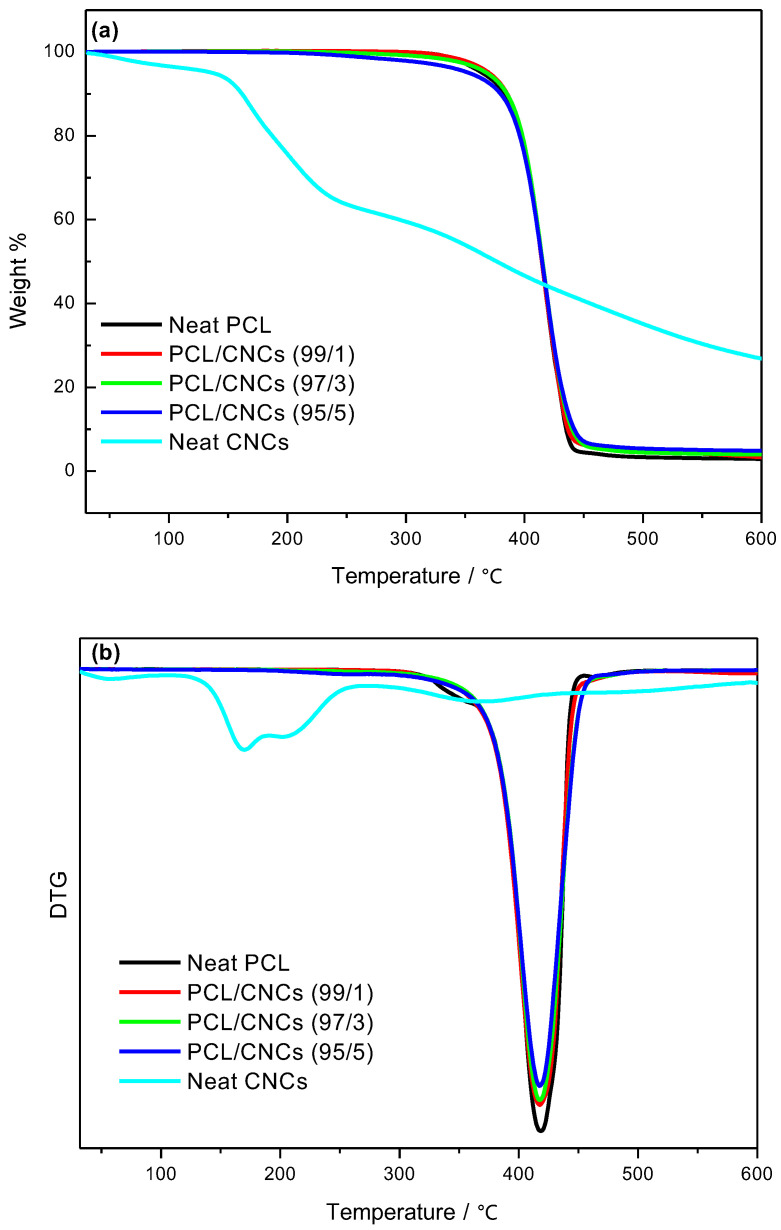
(**a**) TGA and (**b**) DTG curves for neat PCL, neat CNCs and PCL/CNCs nanocomposites with 1, 3 and 5 wt.% CNCs.

**Figure 9:**
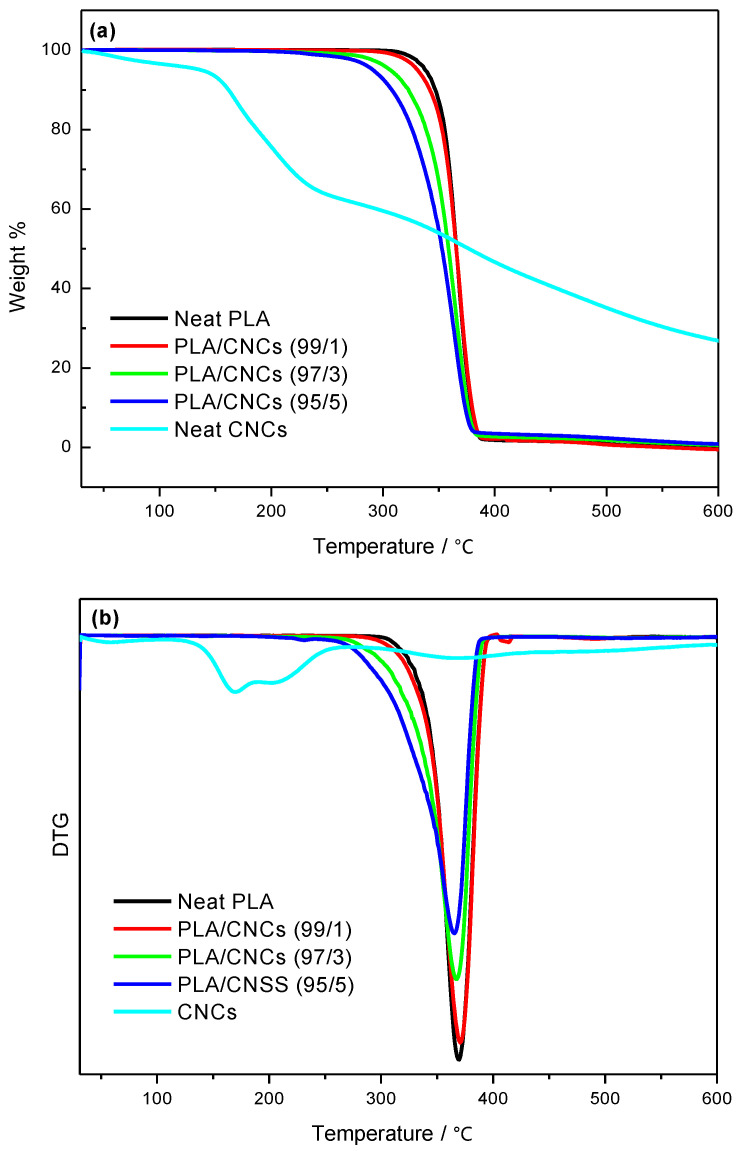
(**a**) TGA and (**b**) DTG curves for neat PLA, neat CNCs, and PLA/CNCs nanocomposites with 1, 3 and 5 wt.% CNCs.

The onset degradation temperature (*T_onset_*), maximum degradation temperature (*T_max_*), and residual weight (*W_residue_*) for neat polymers (i.e. PCL and PLA), neat CNCs and their nanocomposites are summarized in Table 7 and Table 8, while Figure 8 and Figure 9 present the TGA and DTG curves for PCL-based nanocomposites, as well as the PLA-based nanocomposites, respectively. The TGA curve of neat CNCs shows an initial weight loss around 100 °C, attributed to moisture evaporation. Neat CNCs exhibit a two-step degradation process: the first step corresponds to the degradation of sulphated cellulose (sulphate-containing CNCs), while the second step is associated with the breakdown of the interior of non-sulphated crystalline cellulose [65,66]. The *T_onset_*, *T_max_*, and *W_residue_* values for CNCs are reported in Table 7.

The addition of CNCs affects the thermal stability of PCL and PLA nanocomposites differently. For PCL/CNCs nanocomposites (Figure 8a,b), a low CNCs content (1 wt.%) slightly improves thermal stability, as indicated by an increased *T_onset_* compared to neat PCL. However, at higher CNCs loadings (3 and 5 wt.%), thermal stability decreases, likely due to chain disruptions induced by CNCs agglomeration, as reported in Table 8. These results align with previous reports by Hassan et al. and Motloung et al. [12,54].

In PLA/CNCs nanocomposites (Figure 9a,b), both *T_onset_* and *T_max_* decrease with increasing CNCs content (Table 8), reflecting the incorporation of thermally unstable CNCs. Despite this reduction in thermal stability, the presence of CNCs suppresses the escape of volatile degradation products, resulting in an increased char yield of the nanocomposites. These observations are in agreement with the DSC results, which show reduced PLA crystallinity at higher CNCs loadings.

### 3.5. Biodegradation Analysis

**Figure 10:**
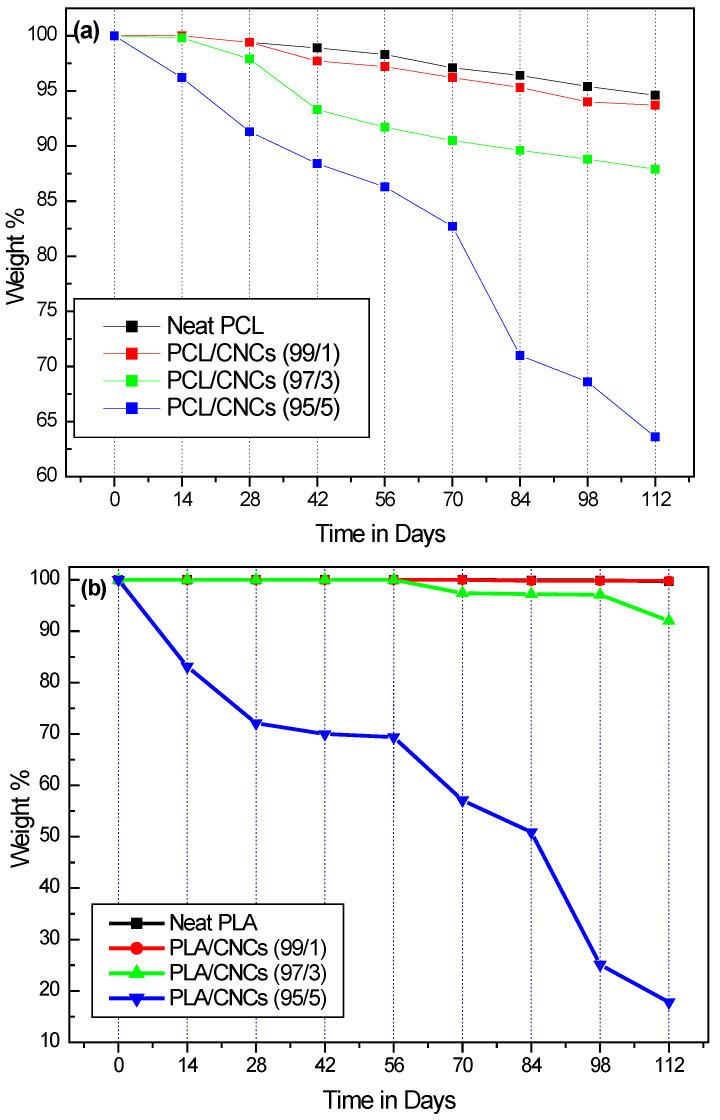
Mass loss (%) as a function of time (days) for (**a**) neat PCL and PCL/CNCs nanocomposite with 1, 3 and 5 wt.% CNCs and (**b**) neat PLA and PLA/CNCs nanocomposites with 1, 3 and 5 wt.% CNCs.

The biodegradation of neat polymers and their nanocomposites was evaluated using a soil burial test, with results presented in Figure 10 and Tables S3 and S4. Degradation patterns for neat PCL and PCL/CNCs nanocomposites are shown in Figure 10a, while those for neat PLA and PLA/CNCs nanocomposites are presented in Figure 10b.

Comparing the neat polymers, PLA degraded by 0.3% over 112 days, whereas PCL exhibited a weight loss of 5.4% during the same period. Degradation rates are influenced by factors such as temperature, moisture, pH, and microbial activity. PLA typically requires higher temperature and humidity, as found in industrial composting conditions [67,68], while PCL degrades faster in soil because of its flexibility, enzyme-accessible structure, low *T_g_*, more permeable crystallinity, and direct susceptibility to microbial attack [69]. Consequently, the soil conditions in this study favored faster degradation of PCL.

At low CNCs concentrations (i.e., 1 wt.%), a uniform dispersion of CNCs was achieved, as confirmed by SEM results. In addition, the crystallinity of PCL and PLA underwent slight alterations, decreasing for PCL and increasing for PLA. However, these changes had a minimal effect on the biodegradation rate. At higher CNCs loading (i.e., 5 wt.%), the crystallinity of PCL increased relative to that of the neat polymer, while the crystallinity of PLA decreased. Notably, the biodegradation rate significantly accelerated, particularly for PLA, which exhibited the fastest degradation, in correlation with its reduced crystallinity. This was observed despite the formation of CNCs aggregates (Figure 4). For PCL, the biodegradation effect was less pronounced, likely due to the increased crystallinity resulting from the nucleating effect of the CNCs. While Hua et al. [70] have demonstrated the importance of CNCs dispersion in influencing the biodegradation rate, it appears that at low concentrations (i.e., 1 wt.%), uniform dispersion alone is insufficient to promote bulk erosion. We therefore conclude that the hydrophilic–hydrophobic balance, which is dependent on CNCs loading, plays a more critical role in determining biodegradation, even when the overall crystallinity of the matrix (i.e., PCL) is increased. This suggests that at low hydrophobic–hydrophilic ratios, both polyesters are more susceptible to hydrolytic cleavage, with the presence of CNCs enhancing the accessibility of the bulk polymer to microbial attack. Table S4 provides digital images of the neat polymers and the nanocomposites samples with varying CNCs content at days 0, 42, 84 and 112, which visually correspond to the quantitative degradation data shown in Figure 10 and Table S3.

## 4. Conclusions

This study demonstrated that cellulose nanocrystals (CNCs) from sugarcane bagasse (SCB-CNCs) can effectively modify the properties of PCL and PLA in a nanocomposite-specific manner. For instance, CNCs had little impact on PCL thermal stability but slightly reduced PLA stability at high loadings (i.e., 5 wt.% CNCs). SEM results showed uniform dispersion of the CNCs at low concentrations; however, agglomerates were seen at high concentrations for both PCL and PLA. CNCs had little impact on the biodegradation rates of PCL and PLA at low at 1 wt.% CNCs, despite the uniform dispersion achieved at this loading. However, fast biodegradation kinetics were observed at 5 wt.% CNCs, owing to the increased hydrophilic character of the nanocomposites, particularly in the case of PLA. This was attributed to decreased crystallinity of PLA at this CNCs concentration, wherein the CNCs decreased the PLA crystallization rate. In contrast, PCL showed limited biodegradability during the same time period, owing to the increased crystallinity, brought about by the nucleating effect of CNCs. The study sought to correlate the crystallinities of the two polyesters with the resultant biodegradation rates, with special emphasis on the dispersion and concentration of CNCs within the matrices. These findings confirm that SCB-derived CNCs can effectively accelerate biodegradation and tune thermal and structural behavior, highlighting their potential as sustainable nanofillers for high-performance, environmentally friendly PCL/PLA materials.

## Figures and Tables

**Table 1:** Sources, yields and properties of CNCs isolated via sulfuric acid hydrolysis from different lignocellulosic biomasses.

Plant Source	Yield (%)	Diameter (nm)	Crystallinity (%)	Reference
SCB	19.3	50	75.9	[current study]
SCB	16.5	20	81.4	[26]
Ramie	-	7.0	90.7	[27]
Jute	57.2 ^a^	55	90.0	[28]
Cotton	42.0	8.0	91.5	[29]
Flax	22.0	10	87.9	[29,30]
Teff straw	93.8 ^a^	31	87.5	[31]
Tobacco	12.5	-	-	[32]
Wood kraft	6.61	9.6	48.2	[33]
Corn stalk	-	6.4	69.2	[34]

^a^ Yield was calculated by authors using cellulose as a reference, not the starting fiber.

**Table 2:** Compositions of PCL, PLA and CNCs in the prepared nanocomposites.

Sample Name	PCL (wt.%)	PLA (wt.%)	CNCs (wt.%)
Neat PCL	100	0	0
Neat PLA	0	100	0
Neat CNCs	0	0	100
PCL/CNCs (99/1)	99	0	1
PCL/CNCs (97/3)	97	0	3
PCL/CNCs (95/5)	95	0	5
PLA/CNCs (99/1)	0	99	1
PLA/CNCs (97/3)	0	97	3
PLA/CNCs (95/5)	0	95	5

Note: PCL = poly(ε-caprolactone); PLA = poly(lactic acid) and CNCs = cellulose nanocrystals.

**Table 3:** The degree of crystallinity results of PCL and PLA in nanocomposites containing 1, 3 and 5 wt.% CNCs.

Sample	PCL *T_c_*/°C & *ΔH_c_*/J/g		PCL *T_m_*/°C & *ΔH_m_*/J/g			PCL *X*_c_/%
	T_c_	ΔH_c_	T_m1_	T_m2_	ΔH_m_	
Neat PCL	18.4 ± 0.0	54 ± 0.1	45.6 ± 0.2	52.2 ± 0.1	56 ± 0.4	40.3
PCL/CNCs (99/1)	21.5 ± 0.0	57 ± 0.1	46.3 ± 0.2	52.5 ± 0.2	48 ± 0.4	34.9
PCL/CNCs (97/3)	22.2 ± 0.0	80 ± 0.7	46.1 ± 0.0	–	74 ± 0.4	54.9
PCL/CNCs (95/5)	19.0 ± 0.0	56 ± 0.3	41.1 ± 0.0	45.2 ± 0.3	64 ± 0.2	48.5

Note: PCL = poly(ε-caprolactone); CNCs = cellulose nanocrystals; *ΔH_m_* = melting enthalpy; *ΔH_c_* = crystallization enthalpy; *ΔH_cc_* = cold crystallization enthalpy; *T_c_* = crystallization temperature; *T*_cc_ = cold crystallization temperature; *T_g_* = glass transition temperature; *T_m_* = melting peak temperature; and *X_c_* = degree of crystallinity.

**Table 4:** DSC results for PLA and PLA/CNCs nanocomposites containing 1, 3, and 5 wt.% CNCs.

Sample	PLA *T_g_/*°C	PLA *T_cc_*/°C & *ΔH_cc_*/J/g		PLA *T_m_*/°C & *ΔH_m_*/J/g			PLA *X_c_*/%
		T_cc_	ΔH_cc_	T_m1_	T_m2_	ΔH_m_	
Neat PLA	59.6 ± 0.1	113.3 ± 0.5	14 ± 1.5	149.1 ± 0.1	–	21 ± 0.2	7.5
PLA/CNCs (99/1)	57.6 ± 0.2	120.1 ± 0.1	21 ± 1.0	149.0 ± 1.1	155.3 ± 0.1	30 ± 0.3	9.7
PLA/CNCs (97/3)	53.6 ± 0.0	115.7 ± 0.0	21 ± 0.8	142.8 ± 0.0	150.4 ± 0.2	24 ± 0.0	3.3
PLA/CNCs (95/5)	46.6 ± 0.1	108.6 ± 0.0	20 ± 1.8	133.6 ± 0.0	142.4 ± 0.3	23 ± 0.4	3.5

Note: PLA = poly(lactic acid); CNCs = cellulose nanocrystals; *ΔH_m_* = melting enthalpy; *ΔH_c_* = crystallization enthalpy; *ΔH_cc_* = cold crystallization enthalpy; *T_c_* = crystallization temperature; *T*_cc_ = cold crystallization temperature; *T_g_* = glass transition temperature; *T_m _* = melting peak temperature; and *X_c_* = degree of crystallinity.

**Table 5:** Crystallinities of neat PCL, PLA, CNCs and their nanocomposites.

Sample	$I_{c}$/%	$(I_{c}$$\;+\;I_{a}$)/%	$X_{cx}$/%
Neat PCL	0.00756	0.0422	17.9
Neat PLA	0.00349	0.0392	8.9
Neat CNCs	0.01265	0.02357	(75.9)
PCL/CNCs (99/1)	0.00651	0.03871	16.8
PCL/CNCs (97/3)	0.0140	0.06556	21.4
PCL/CNCs (95/5)	0.00399	0.0237	16.8
PLA/CNCs (99/1)	–	0.06553	–
PLA/CNCs (97/3)	–	0.06575	–
PLA/CNCs (95/5)	–	0.05862	–

Note: PCL = poly(ε-caprolactone); PLA = poly(lactic acid); CNCs = cellulose nanocrystals.

**Table 6:** SAXS parameters for neat PCL, PLA, CNCs and their nanocomposites with 1, 3 and 5 wt.% CNCs loading.

Sample	$q_{max}$/nm^−1^	*L*/nm	$l_{c}$/nm.%
Neat PCL	0.40	15.1	270.3
Neat PLA	0.29	21.7	193.1
Neat CNCs	–	–	–
PCL/CNCs (99/1)	0.44	14.3	240.2
PCL/CNCs (97/3)	0.46	13.7	293.2
PCL/CNCs (95/5)	0.48	13.1	220.1
PLA/CNCs (99/1)	–	–	–
PLA/CNCs (97/3)	–	–	–
PLA/CNCs (95/5)	–	–	–

Note: PCL = poly(ε-caprolactone); PLA = Poly(lactic acid); CNCs = cellulose nanocrystals.

**Table 7:** Thermal characteristics of CNCs, including onset temperature, peak temperature and residual weight.

Sample	*T*_1*onset*_/°C	*T*_1*max*_/°C	*T*_2*onset*_/°C	*T*_2*max*_/°C	*W_residue_*/%
CNCs	128.4	168.8	299.4	368.8	26.9

Note: CNCs = cellulose nanocrystals; *T_onset_* = onset temperatures; *T_max_* = peak temperatures; *W_residue_* = residual weight.

**Table 8:** Thermal stabilities of neat PCL, PLA and their nanocomposites.

Sample	PCL *T_onset_*/°C	PCL *T_max_*/°C	PLA *T_onset_*/°C	PLA *T_max_*/°C	*W_residue_*/%
Neat PCL	298.9	418.0	−	−	3.1
PCL/CNCs (99/1)	310.3	417.9	−	−	3.3
PCL/CNCs (97/3)	289.6	417.9	−	−	3.9
PCL/CNCs (95/5)	243.1	417.9	−	−	4.8
Neat PLA	−	−	305.8	369.0	0.0
PLA/CNCs (99/1)	−	−	282.8	371.0	0.0
PLA/CNCs (97/3)	−	−	259.0	366.8	0.4
PLA/CNCs (95/5)	−	−	218.6	364.7	1.0

Note: PCL = poly(ε-caprolactone); PLA = poly(lactic acid) and CNCs = cellulose nanocrystals; *T_onset_* = onset temperatures; *T_max_* = peak temperatures; *W_residue_* = residual weight.

## Data Availability

The original contributions presented in this study are included in the article/supplementary material. Further inquiries can be directed to the corresponding author(s).

## Supplementary Materials

The following supporting information can be downloaded at: https://www.mdpi.com/article/10.3390/polym18091132/s1, Figure S1: Extraction of cellulose and CNCs from SCB; Figure S2: Melting temperatures of (a) PCL and (b) PLA with 1, 3 and 5 wt.% CNCs; Figure S3: SAXS plots for Intensity (*I*) versus scattering vector (*q*) for (a) neat PCL, neat CNCs and PCL/CNCs nanocomposites with 1, 3 and 5 wt.% CNCs, and (b) neat PLA, neat CNCs and PLA/CNCs nanocomposites with 1, 3 and 5 wt.% CNCs (data obtained from the second heating at 25 °C); Table S1: Extraction yields of cellulose and CNCs obtained from SCB; Table S2: Drying conditions for PCL, PLA and CNCs; Table S3: Biodegradation analysis shows mass loss (%) versus time (days) for neat PCL and PCL/CNCs nanocomposites, and for neat PLA and PLA/CNCs nanocomposites; Table S4: Biodegradation digital images of neat polymers and their nanocomposites containing 1, 3 and 5 wt.% CNCs, captured on days 0, 42, 84 and 112.

## Abbreviations

The following abbreviations are used in this manuscript:

CNCs	Cellulose nanocrystals
DSC	Differential scanning calorimetry
FTIR	Fourier transform infrared spectroscopy
H_2_SO_4_	Sulphuric acid
NaOH	Sodium hydroxide
PCL	Poly(ε-caprolactone)
PLA	Poly(lactic acid)
SAXS	Small angle x-ray scattering
SCB	Sugarcane bagasse
SEM	Scanning electron microscopy
TGA	Thermogravimetric analysis
WAXS	Wide angle x-ray scattering
