# Supplementary material for: Effect of Sugarcane Bagasse-Derived Cellulose Nanocrystals on the Thermal, Structural, Morphological and Biodegradation Properties of Poly(ε-caprolactone) and Poly(lactic Acid)
Source: Polymers (Basel). 2026 May 4;18(9):1132. doi: 10.3390/polym18091132 (PMC13165788; doi:10.3390/polym18091132)
Supplement: Supplementary file 1 [file polymers-18-01132-s001.zip › polymers-4165862-supplementary.pdf]

Supporting Information

Effect of Sugarcane Bagasse-Derived Cellulose Nanocrystals on the Thermal, Structural, Morphological and Biodegradation Properties of Poly(ε-caprolactone) and Poly(lactic Acid)

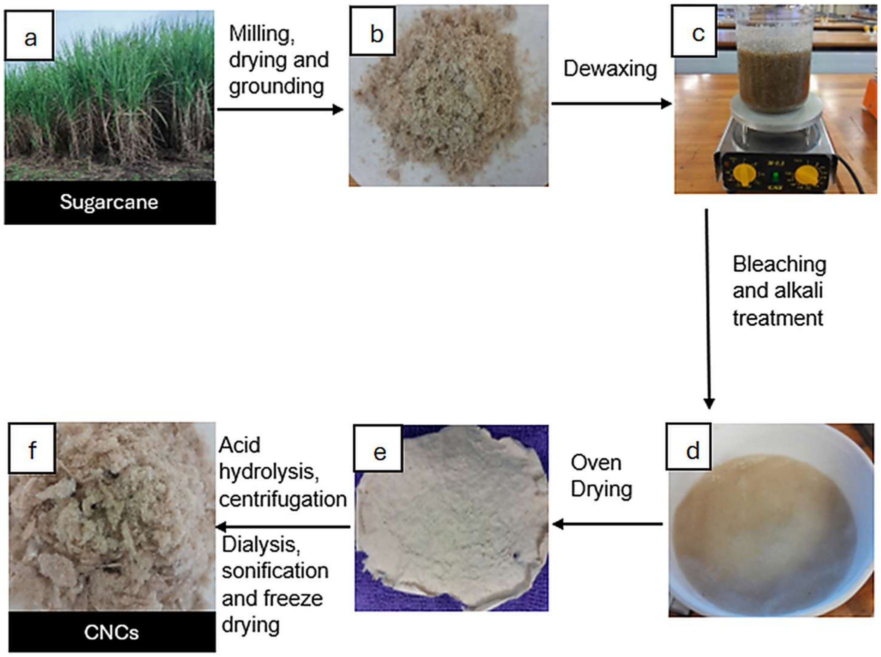

Figure S1. Extraction of cellulose and CNCs from SCB.

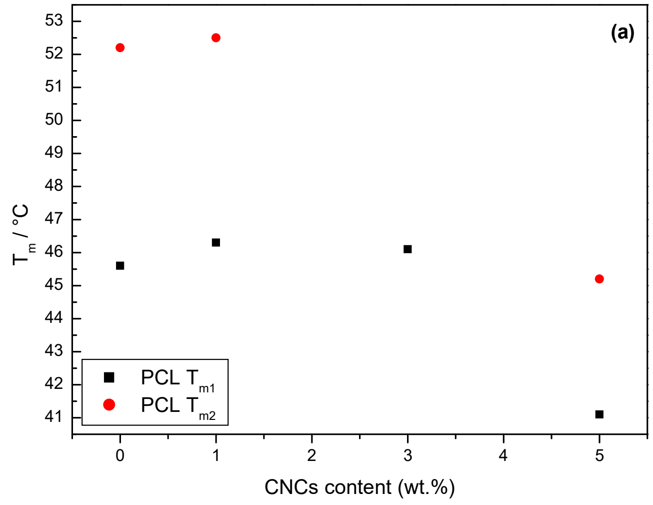

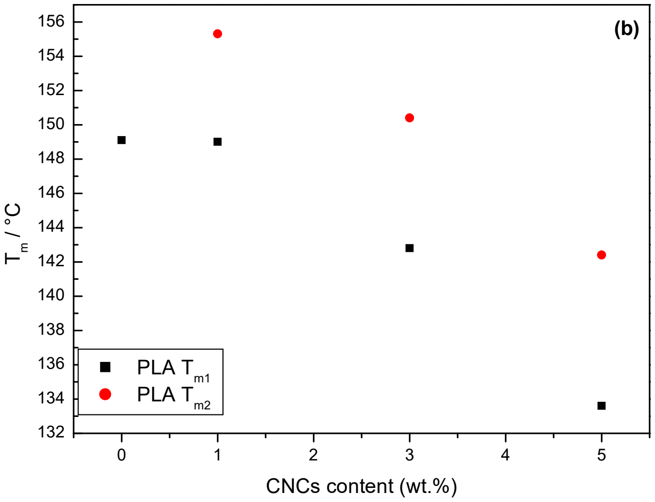

Figure S2. Melting temperatures of (a) PCL and (b) PLA with 1, 3 and 5 wt.% CNCs .

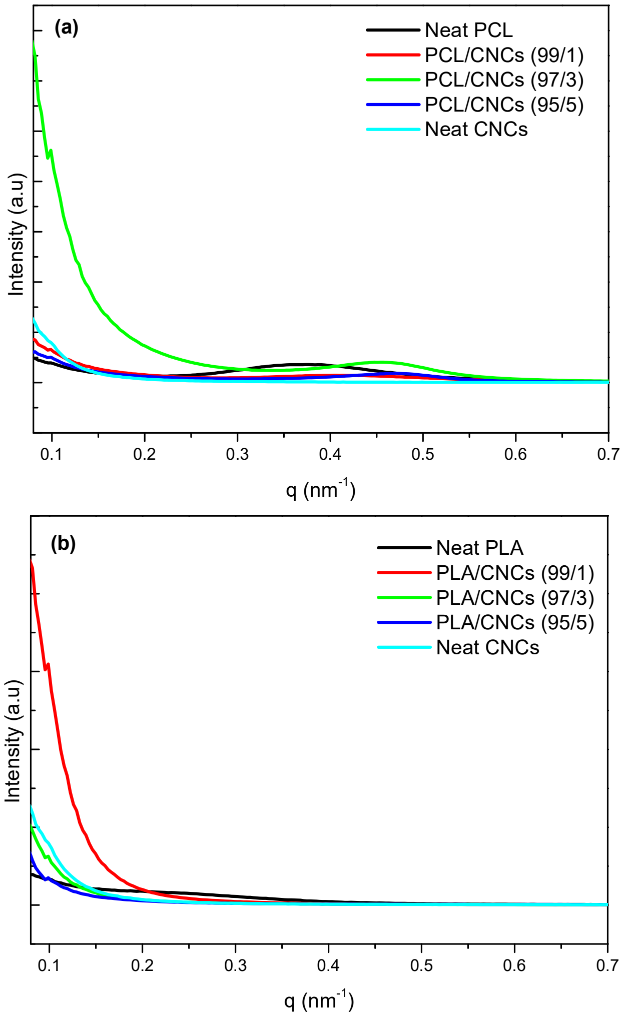

Figure S3. SAXS plots for Intensity (*I*) versus scattering vector (*q*) for (a) neat PCL, neat CNCs and PCL/CNCs nanocomposites with 1, 3 and 5 wt.% CNCs, and (b) neat PLA, neat CNCs and PLA/CNCs nanocomposites with 1, 3 and 5 wt.% CNCs(data obtained from the second heating at 25 °C).

Table S1. Extraction yields of cellulose and CNCs obtained from SCB**.**

Sample	Mass of the ground powder (g)	Mass of cellulose (g)	% yield of cellulose	Mass of CNCs (g)	% yield of CNCs
SCB	40	10.5	26.3	7.7	19.25

Note: CNCs = Cellulose nanocrystals and SCB = sugarcane bagasse

Table S2. Drying conditions for PCL, PLA and CNCs.

Material	Drying conditions
PCL	40 °C, in vacuum oven, overnight
PLA	50 °C, in vacuum oven, overnight
CNCs	50 °C, in vacuum oven, overnight

Table S3. Biodegradation analysis shows mass loss (%) versus time (days) for neat PCL and PCL/CNCs nanocomposites, and for neat PLA and PLA/CNCs nanocomposites.

Sample	Day 0	Day 14	Day 28	Day 42	Day 56	Day 70	Day 84	Day 98	Day 112
Neat PCL	100	100	99.4	98.9	98.3	97.1	96.4	95.4	94.6
PCL/CNCs (99/1)	100	100	99.4	97.7	97.2	96.2	95.3	94.0	93.7
PCL/CNCs (97/3)	100	99.8	97.9	93.3	91.7	90.5	89.6	88.8	87.9
PCL/CNCs (95/5)	100	96.2	91.3	88.4	86.3	82.7	71.0	68.6	63.6
Neat PLA	100	100	100	100	100	100	99.9	99.9	99.7
PLA/CNCs (99/1)	100	100	100	100	100	100	99.8	99.8	99.8
PLA/CNCs (97/3)	100	100	100	100	100	97.4	97.2	97.1	92.0
PLA/CNCs (95/5)	100	83.1	72.1	70.0	69.4	57.1	50.9	25.1	17.8

Note: PCL = poly(ε-caprolactone); PLA = poly(lactic acid) and CNCs = cellulose nanocrystals

Table S4. Biodegradation digital images of neat polymers and their nanocomposites containing 1, 3 and 5 wt.% CNCs, captured on days 0, 42, 84 and 112.

**Day 0**		**Day 42**		**Day 84**		**Day 112**	
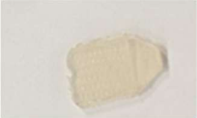<br>Neat PLA	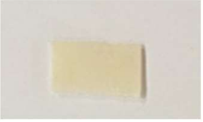<br>Neat PCL	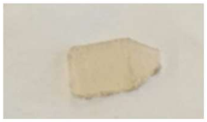<br>Neat PLA	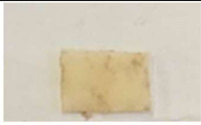<br>Neat PCL	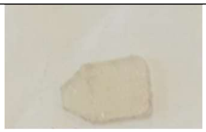<br>Neat PLA	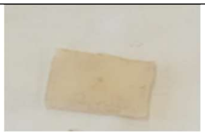<br>Neat PCL	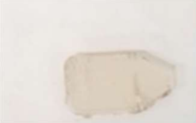<br>Neat PLA	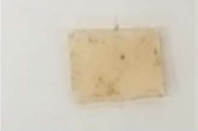<br>Neat PCL
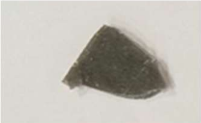<br>PLA/CNCs 99/1	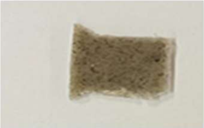<br>PCL/CNCs 99/1	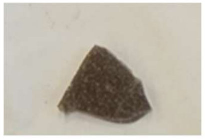<br>PLA/CNCs 99/1	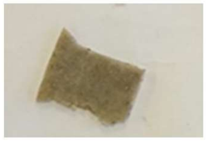<br>PCL/CNCs 99/1	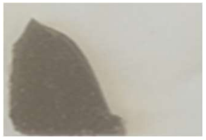<br>PLA/CNCs 99/1	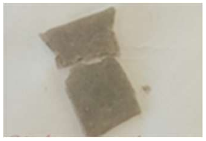<br>PCL/CNCs 99/1	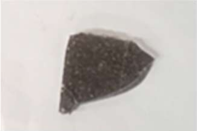<br>PLA/CNCs 99/1	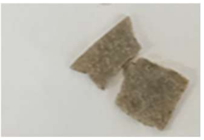<br>PCL/CNCs 99/1
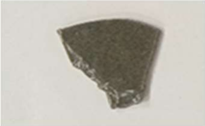<br>PLA/CNCs 97/3	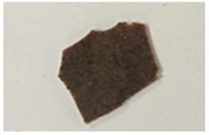<br>PCL/CNCs 97/3	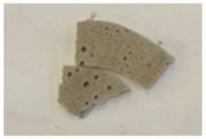<br>PLA/CNCs 97/3	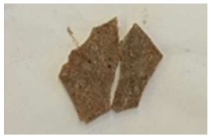<br>PCL/CNCs 97/3	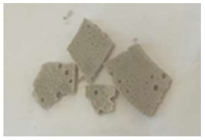<br>PLA/CNCs 97/3	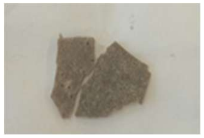<br>PCL/CNCs 97/3	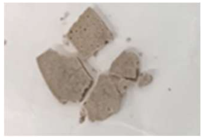<br>PLA/CNCs 97/3	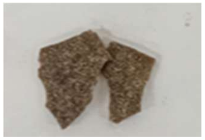<br>PCL/CNCs 97/3
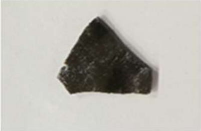<br>PLA/CNCs 95/5	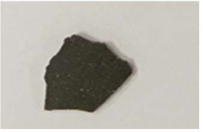<br>PCL/CNCs 95/5	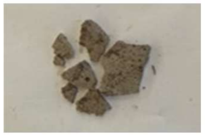<br>PLA/CNCs 95/5	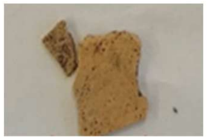<br>PCL/CNCs 95/5	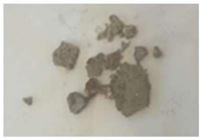<br>PLA/CNCs 95/5	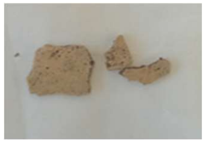<br>PCL/CNCs 95/5	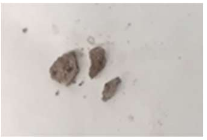<br>PLA/CNCs 95/5	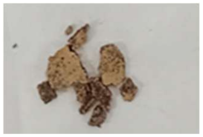<br>PCL/CNCs 95/5
